# Network Based Consensus Gene Signatures for Biomarker Discovery in Breast Cancer

**DOI:** 10.1371/journal.pone.0025364

**Published:** 2011-10-25

**Authors:** Holger Fröhlich

**Affiliations:** University of Bonn, Bonn-Aachen International Center for IT (B-IT), Bonn, Germany; Institute of Molecular and Cell Biology, Singapore

## Abstract

Diagnostic and prognostic biomarkers for cancer based on gene expression profiles are viewed as a major step towards a better personalized medicine. Many studies using various computational approaches have been published in this direction during the last decade. However, when comparing different gene signatures for related clinical questions often only a small overlap is observed. This can have various reasons, such as technical differences of platforms, differences in biological samples or their treatment in lab, or statistical reasons because of the high dimensionality of the data combined with small sample size, leading to unstable selection of genes. In conclusion retrieved gene signatures are often hard to interpret from a biological point of view. We here demonstrate that it is possible to construct a consensus signature from a set of seemingly different gene signatures by mapping them on a protein interaction network. Common upstream proteins of close gene products, which we identified via our developed algorithm, show a very clear and significant functional interpretation in terms of overrepresented KEGG pathways, disease associated genes and known drug targets. Moreover, we show that such a consensus signature can serve as prior knowledge for predictive biomarker discovery in breast cancer. Evaluation on different datasets shows that signatures derived from the consensus signature reveal a much higher stability than signatures learned from all probesets on a microarray, while at the same time being at least as predictive. Furthermore, they are clearly interpretable in terms of enriched pathways, disease associated genes and known drug targets. In summary we thus believe that network based consensus signatures are not only a way to relate seemingly different gene signatures to each other in a functional manner, but also to establish prior knowledge for highly stable and interpretable predictive biomarkers.

## Introduction

Diagnostic and prognostic biomarkers for cancer based on patient gene expression profiles are viewed as a major step towards a better personalized medicine. Identification of disease-subtypes and risk stratification of patients based on specific biomarker gene signatures has the potential to help medical doctors to find an individually optimized treatment, to avoid unnessery medication and to reduce costs [1]–[3].

A wealth of gene expression data for patients is nowadays publicly available through databases such as Gene Expression Omnibus [4], [5], ArrayExpress [6] and The Cancer Genome Atlas (TCGA). Recently, GeneSigDB [7] has been established as a database systematically integrating gene signatures (i.e. lists of genes being together associated with a certain phenotype) from various publications leading to a rich resource for meta analysis and high level comparisons. Several authors have mentioned the small overlap when comparing gene signatures from different studies [8]–[10]. This imposes a difficulty for interpretation and validation of gene signatures, since in general biomarker research has two major goals: first, identification of stable and robust disease markers (i.e. molecules, which are causally linked to the disease phenotype), and second, discovery of targets for potential therapies. For both purposes biomarkers are required to be reproducible [11].

Major differences between gene signatures for related disease phenotypes can in principal have various reasons [12]: (1) Different chip platforms could have been used, which may imply non-identical sets of measured transcripts and thus can lead to systematic differences in obtained gene signatures. (2) The biological material may show certain systematic differences between microarray studies. If, for example, breast cancer patients in one study have a higher average age than in another study, this may lead to differences in gene expression. (3) Experimental protocols may differ between laboratories. (4) Microarray data is very high dimensional and thus establishing a statistically stable gene signature is a severe problem in the light of usual small sample sizes. Depending on the chosen technique, minimal changes of dataset 1 versus dataset 2 may thus lead to drastic changes of obtained gene signatures.

Despite of this fact it was found that on a functional level (e.g. dis-regulated pathways) gene signatures with small overlap can be rather similar [9], [13]. This motivated us to investigate two questions: (1) Is it possible to derive a *consensus signature* from a set of published prognostic gene signatures and does this consensus signature exhibit functional enrichment of disease related genes and known drug targets? (2) Can this consensus signature be used as prior knowledge to derive predictive gene signatures, which have a high stability and are easy to interprete?

To derive a consensus signature we here propose an algorithm, which clusters genes from different gene signatures based on their shortest path distances in a protein-protein interaction network. For each cluster we then identify so-called lowest-common ancestors (LCAs), which are proteins that are commonly upstream of a set of proteins and thus may exhibit a certain regulatory influence (provided they are not too far away). This idea has a certain similarity with the master regulator analysis (MRA) algorithm proposed in a different context for reconstruction of disease specific TF-target networks [14]. The set of LCAs, eventually joined with the set of genes appearing in the majority of signatures, forms our consensus signature. Our hypothesis, which we verified here by looking at six signatures related to breast cancer prognosis [15]–[20], was that genes in such a consensus are enriched for genes that are known to be disease associated. Moreover, we found a strong enrichment of known drug targets.

Having verified this first hypothesis we went on to test our second hypothesis, namely that genes in the consensus signature can be used to guide development of predictive gene signatures in breast cancer, which are interpretable and stable. This hypothesis was verified in three independent gene expression datasets [21]–[23]. These derived signatures not only showed a significant overlap, but were also more stable with respect to selected genes than signatures learned from the full set of probesets on a microarray. Furthermore, signatures derived from the consensus signature showed a high fraction of disease related genes and targets for therapeutic compounds, which highlights the possibility to interpret them easily.

## Results

### Network Based Consensus Signature is Disease Related and Enriched for Known Drug Targets

Six gene signatures related to breast cancer prognosis, namely [15]–[20], were retrieved in standardized format (ENSEMBL identifiers) from GeneSigDB. An overview about these signatures is given in Table 1. We converted signatures to Entrez gene IDs and mapped them to a large protein interaction network compiled from a merger of the Pathway Commons database [24] with non-metabolic KEGG pathways (see Section Methods). These six signatures together contained 504 genes, among which 28 appeared more than once and 17 three times. There was no overall overlap between signatures, and no gene appearing in more than half of the signatures.

**Table 1:** Overview about used gene signatures.

Signature	PMID	predicts	#Patients	#Entrez IDs
Bertucci et al.	12538167	long vs. short term survival	34	21
Li et al.	18278552	recurrence	93	28
Huang et al.	12747878	recurrence	89	148
Van't Veer et al.	11823860	long vs. short term survival	117	60
Wang et al.	15721472	metastasis occurrence	115	75
Sotiriou et al.	16478745	histologic grade	189	223

The last column shows the number of *unique* Entrez Gene IDs.

An algorithm was developed for deriving a network based consensus signature from these six signatures consisting of lowest common anchestors (LCAs). Details of the algorithm as well as a computational study investigating features of its principal performance are described in the Methods Section of this paper. Application of this algorithm yielded a consensus signature with 203 genes (Table S1), which was investigated further.

**Figure 1:**
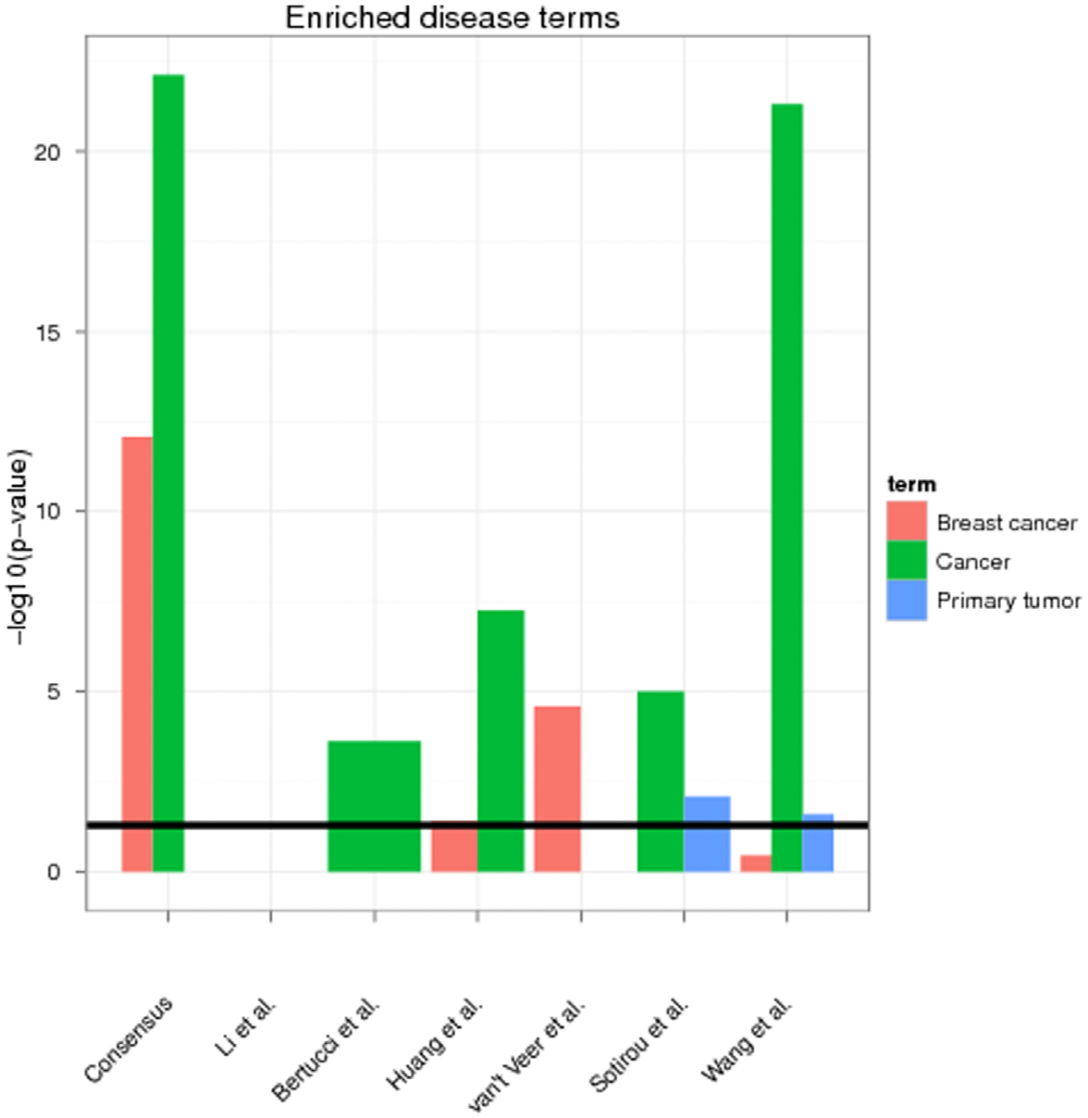
Enrichment of “cancer”, “breast cancer” and “primary tumor” related genes in consensus and original signatures (hypergeometric test). Only Disease Ontology terms are depicted, which map to at least 2 genes from a signature. The black line indicates a 5% significance threshold (after Bonferroni correction). The full list of all enriched Disease Ontology terms with Bonferroni corrected p<5% is available in Table S1.

**Figure 2:**
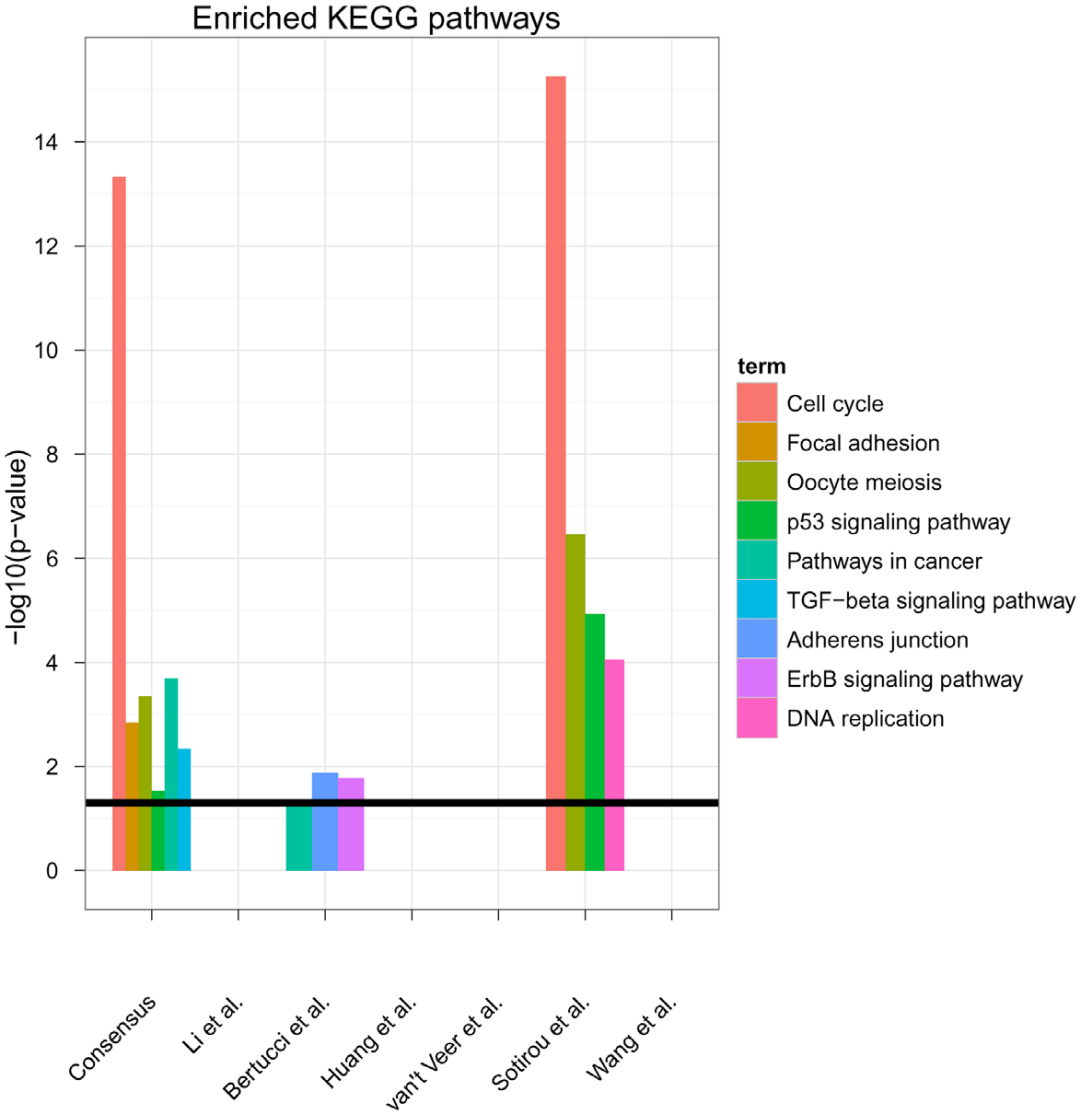
Enrichment of cancer related pathways in consensus and original signatures (hypergeometric test). Only signficantly enriched pathways are depicted. The black line indicates a 5% FDR significance threshold (Benjamini-Yekutieli method). The full list of all enriched KEGG pathways with FDR<5% is available in Table S3.

We used the tool FunDO [25] to look for enrichment of disease related genes (Table S2). FunDO uses a hypergeometric test. This revealed a high enrichment of cancer (36 genes, Bonferroni corrected p<1e-22) and specifically breast cancer related genes (21 genes, Bonferroni corrected p<1e-12). Interestingly enough the enrichment of cancer and breast cancer genes was even higher in the consensus signature than in the original signatures (Figure 1). A further analysis of enriched KEGG pathways (hypergeometric test, Figure 2) also showed a high enrichment of “Cell cycle” (FDR<1e-13, multiple testing correction by Benjamini-Yekutieli method [26]), “Pathways in cancer” (FDR<0.001), “TGF-*β* signaling pathway” (FDR<0.01), “Focal adhesion” (FDR<0.01), “Oocyte meiosis” (FDR<0.001) and “p53 signaling pathway” (FDR<0.05), which have all been related to breast cancer [27]–[30]. Interestingly, the only other signature showing such a strong enrichment of cancer related KEGG pathways was that of Sotiriou et al., whereas the signature by Bertucci et al. showed a much weaker enrichment (“ErbB signaling”, “Pathways in cancer”, “Adheres junction” with FDR<0.05) and the others revealed no enrichment at FDR cutoff 5%. In other words, no commonly enriched pathways could be found among the six published signatures investigated here.

**Figure 3:**
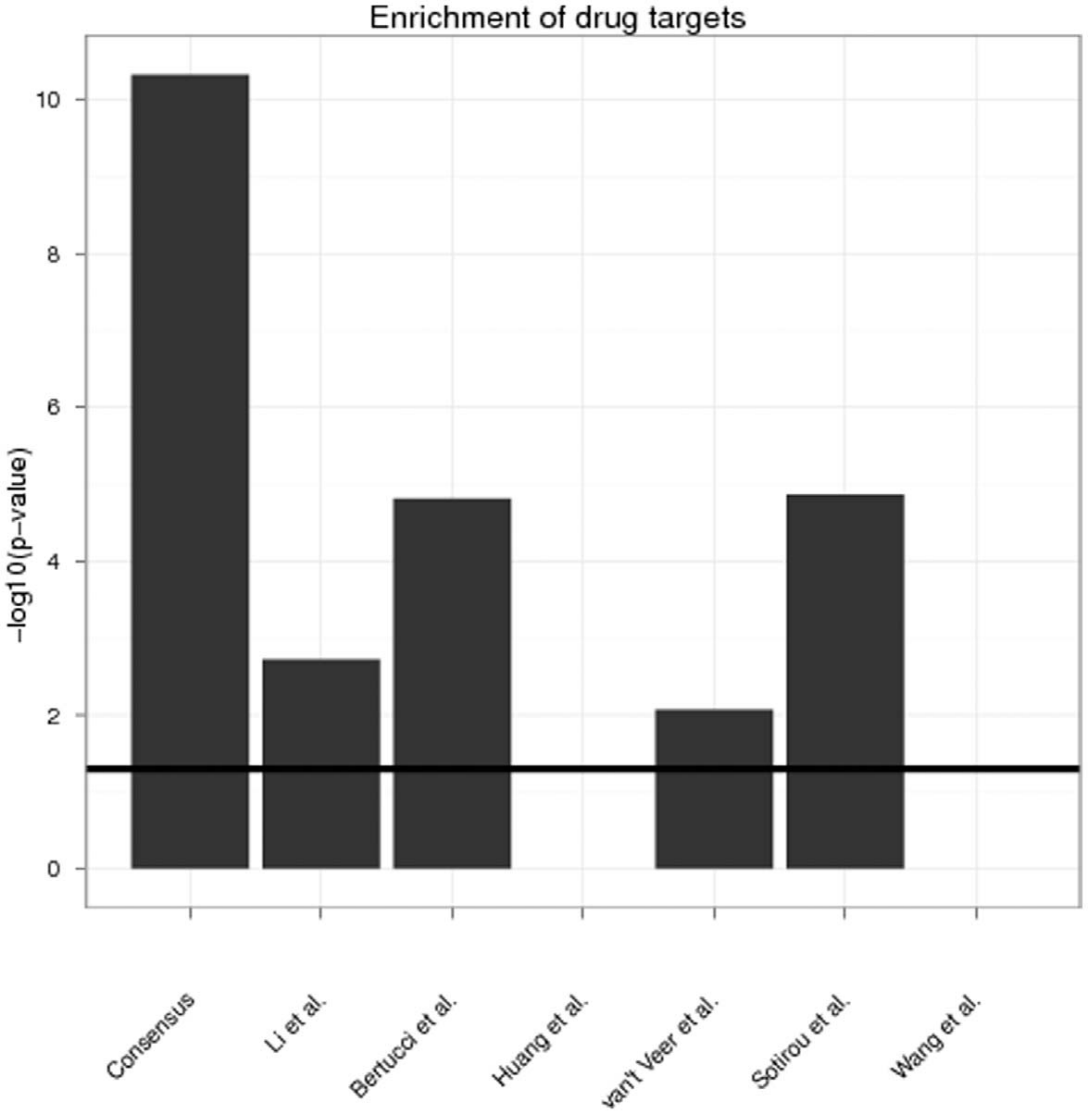
Enrichment of drug targets in consensus and original signatures (hypergeometric test). The black line indicates a 5% significance threshold. The full list of all drug targets and therapeutic compounds is available in Table S4.

We finally looked for an enrichment of targets for therapeutic compounds against breast cancer in our consensus signature. For that purpose we retrieved a list of 104 proteins and respective therapeutic compounds in breast cancer, which are either in clinical trials (also withdrawn ones), FDA approved or on the market with the help of the software MetaCore (see Supplement S1 and Table S4). Application of Fisher's exact test revealed a high over-representation of such drug targets within our consensus signature (p<1e-10). This was interestingly higher than for all other signatures (Figure 3). Signatures by Wang et al. and Huang et al. did not show any enrichment of known drug targets.

### Network Based Consensus Signature Can Guide Predictive Biomarker Development

#### Microarray Data

We next sought out to investigate, in how far a network based consensus signature can be used to guide development of prognostic gene signatures. For that purpose we took three independent microarray datasets, which had not been used to establish any of our above investigated signatures. These were the data by Ivshina et al. [21] (249 patients), Schmidt et al. [22] (“Mainz dataset”; 200 patients) and Pawitan et al. [23] (159 patients). All data were measured on the Affymetrix HGU133A chip platform, downloaded from GEO and normalized via FARMs [31].

#### Predictive Power and Stability

We used SAM [32] to identify probesets being differentially expressed between different clinical groups of patients with a q-value cutoff of 5%. Depending on the available information at GEO for each dataset these groups were defined slightly different: On the Mainz dataset we tried to discriminate patients with distant metastasis-free survival <5 years (46 patients) from patients with longer distant metastasis-free survival (154 patients). On the Ivshina and Pawitan datasets we looked for differentially expressed probesets between patients suffering from a disease recurrence within 5 years (Ivshina: 90, Pawitan: 40) from those, with longer relapse-free survival (Ivshina: 159, Pawitan: 119). Only probesets corresponding to genes in our consensus signature were used. Given these probesets we then trained a Support Vector Machine (SVM) with linear kernel and hyperparameter *C* tuned in the range 10^−4^,10^−3^,…10^2^ via 5-fold cross-validation.

**Figure 4:**
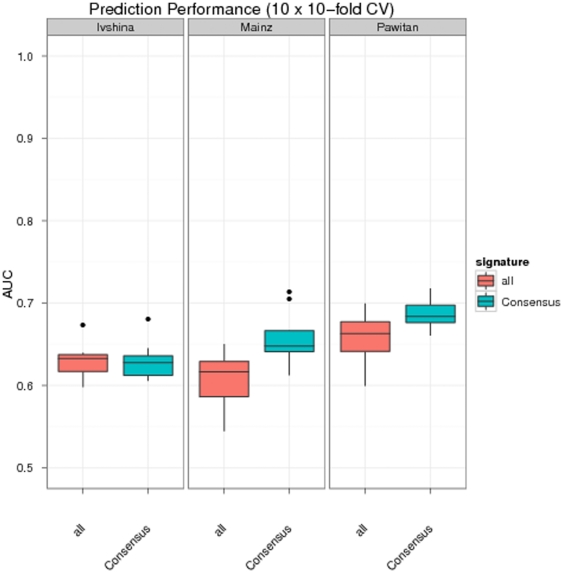
Prediction performance of differentially expressed probesets within our consensus signature (*Consensus*). *All* refers to a signature derived from all probesets on the microarray. All signatures were trained and tested within a cross-validation procedure.

We evaluated the prediction performance of this approach within a cross-validation scheme on each of the above described datasets: Each dataset was randomly split into 10 folds and each fold successively left out once for testing, while the others were used for training (SAM analysis plus subsequent SVM training). The whole procedure was repeated 10 times (10 times repeated 10-fold cross-validation). Predictions were then evaluated in terms of area under ROC curve (AUC). This showed a performance of our approach being significantly better (Mainz and Pawitan datasets, p<0.05-Wilcoxon signed rank test) or at least comparable (Ivshina dataset) as if differentially expressed probesets among all probesets on the microarray were identified and used for SVM training (Figure 4). That means our consensus signature was not only interpretable, but also contained a predictive signal.

**Figure 5:**
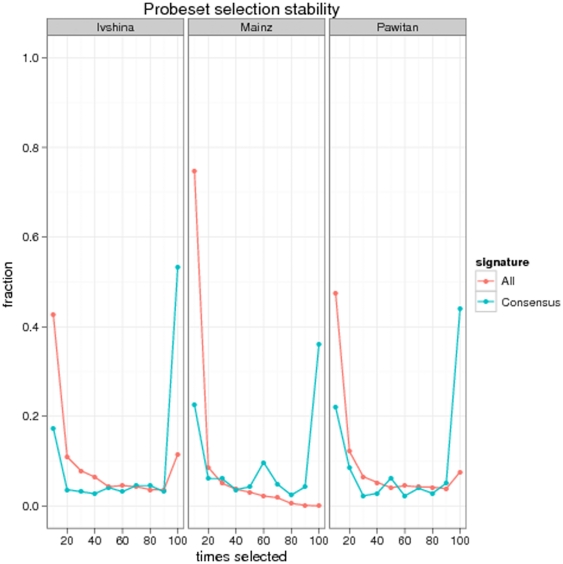
Stability of probeset selection: The x-axis shows the number of times a probeset is selected within a 10×10-fold cross-validation procedure (i.e. 100 times at maximum). The y-axis depicts the fraction of probesets, which have been selected as often as indicated on the x-axis.

An analysis of the frequency, by which individual probesets were selected within the cross-validation procedure clearly revealed a higher stability compared to a signature constructed from all probesets on the microarray (Figure 5): On the Ivshina dataset the fraction of constantly chosen probesets was 50% among all those that were ever selected. On the Pawitan and Mainz dataset the fraction was around 40%. In contrast, learning a signature from all probesets on the microarray yielded only a fraction of ^∼^10% constantly chosen probesets on the Ivshina and below 5% on the other datasets. This result is not unexpected: Most genes are unable to perfectly separate patient groups individually. Then, depending on the selected set of patients, they may sometimes appear as significant, sometimes not, which makes gene selection unstable. Therefore, it is natural that restricting the set of probesets by some sort of prior knowledge (as we did here) increases gene selection stability. Our approach may thus be understood as a specific kind of regularization [33], in which model complexity (here: maximal number of genes in a gene signature, which is upper bounded by the size of the consensus signature) is restricted in order to increase the possibility to identify a good fitting classifier with high stability. The whole idea can be illustrated further by a simulation, in which we compared stabilities of randomly selected signatures from the whole microarray against randomly selected signatures from an a-priori defined set of probesets (see detailed description in Supplement S1 and Figure S1).

#### Functional Analysis

We further had a closer look at the individual signatures derived from the consensus signature (Tables S5, S6, S7). For that purpose we ran a SAM analysis on each of the three microarray datasets without cross-validation, only using probesets of our consensus signature. This yielded 111 genes (175 probesets) for the Ivshina data set, of which FunDO could relate 26 to cancer (Bonferroni corrected p<1e-19, see Table S7) and 10 to breast cancer (Bonferroni corrected p<1e-4). Moreover, the signature contained 7 known targets for therapeutic compounds (enrichment: p<2.2e-16), namely *HSP90AA1*(e.g. Retaspimycin, Tanespimycin, Alvespimycin – see Table S5), *TOP2A* (e.g. Esorubicin, Ciprofloxacin), *ERBB2* (e.g. Trastuzumab, Pertuzumab, Gefitinib), *TUB4A1A* (e.g. Estramustine), *TUBA1B* (e.g. Eribulin, Paclitaxel), *CDK2* (e.g. Indisulam) and *EGFR* (e.g. Erlotinib, Gefitinib). KEGG analysis revealed a high enrichment of “Cell cycle” (FDR<1e-10), “Progesterone-mediated oocyte maturation” (FDR<0.001), “Oocyte meiosis” (FDR<0.01) and “Pathways in cancer” (FDR<0.01).

For the Mainz dataset we obtained 69 genes (101 probesets), of which FunDO could relate 18 to cancer (Bonferroni corrected p<1e-14) and 3 specifically to breast cancer. Moreover, the signature contained 3 known targets for therapeutic compounds (enrichment: p<1e-4), namely *TOP2A*, *CDK2* and *TUBA1B*. KEGG analysis revealed an enrichment of “Cell cycle” (FDR<1e-10), “Progesterone-mediated oocyte maturation” (FDR<0.01), “Oocyte meiosis” (FDR<0.01) and “p53 signaling pathway” (FDR<0.05).

Analysis of the Pawitan dataset yielded 78 genes (113 probesets), of which FunDO could relate 19 to cancer (Bonferroni corrected p<1e-14), 2 to primary tumors (Bonferroni corrected p<0.05) and 8 specifically to breast cancer (Bonferroni corrected p<1e-4). The signature contained 4 known targets for therapeutic compounds (enrichment: p<1e-14), namely *HSP90AA1*, *TOP2A*, *TUBA1B* and *CDK2* (Table S7). Enriched KEGG pathways were again “Cell cycle” (FDR<1e-9), “Progesterone-mediated oocyte maturation” (FDR<0.001) and “Oocyte meiosis” (FDR<0.001).

**Figure 6:**
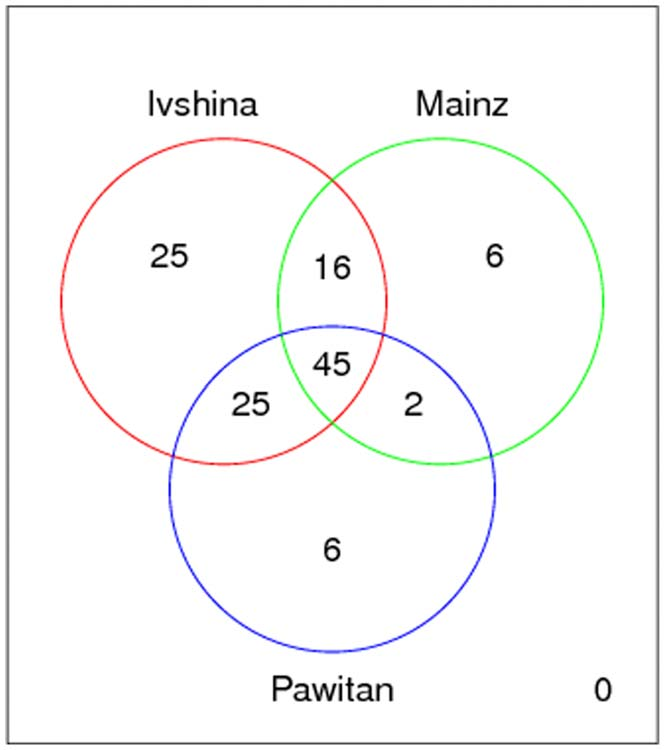
Overlap of signatures derived from the consensus signature. The total overlap between all three signatures was significant with p<1e-5.

Altogether these results revealed that predictive biomarker signatures derived from the consensus signature, showed a clear disease association in terms of enriched pathways, therapeutic targets and disease related genes. Moreover, there was a high consistency in enriched pathways, namely “Cell Cycle”, “Progesterone-mediated oocyte maturation” and “Oocyte meiosis”. Even at the gene level we observed a significant overlap (Figure 6; p<1e-5). Significance was determined here via a permutation test (see Methods).

The results presented in this paragraph generally met our expectations, since all signatures were derived from the consensus signature, which has already been shown to be highly enriched for cancer related pathways, disease related genes and therapeutic drug targets.

## Discussion

In this work we demonstrated that it is possible to derive a consensus signature from seemingly different prognostic gene signatures in breast cancer by taking into account knowledge on protein-protein interactions. Our approach is based on the idea, that genes from different signatures can be clustered in the context of a protein interaction network and that meaningful representatives for these clusters can be found by looking for close, common upstream genes. Application of this method to six published gene signatures and subsequent enrichment analysis revealed a clear association of our consensus signature to breast cancer related genes, pathways and targets for therapeutic compounds.

We demonstrated that network based consensus signatures can be useful as prior knowledge for prognostic biomarker discovery. We suppose that the same framework could likewise be used in the context of diagnostic biomarker discovery (disease subtype identification). On our investigated datasets the cross-validated prediction performance with our approach, where only differentially expressed probesets within the consensus signature were considered, was at least comparable as if differentially expressed probesets from the whole microarray were taken into account. Moreover and most importantly, gene selection stability was significantly higher. The retrieved final signatures were meaningful in terms of enriched pathways, drug targets and breast cancer related genes.

In summary we thus believe that looking for consensus signatures among published gene signatures from the literature is not only a possibility to establish a functional relationship between these signatures, but also offers a valuable source of prior knowledge for biomarker discovery in breast cancer and thus can bring us a bit closer to the ultimate goal to obtain an interpretable, stable and highly predictive gene signature for patient stratification according risk groups and disease subtypes. It remains, however, an open question for future work, in how far our presented results can be generalized to other disease entities.

## Materials and Methods

### Protein Interaction Network

Within this work we employ protein interaction data as our basic knowledge resource. In our case a protein interaction network was compiled from a merger of all non-metabolic KEGG pathways [34] – only gene-gene interactions were considered – together with the Pathway Commons database [24], which was downloaded in tab-delimited format (May 2010). The purpose was to obtain an as much as possible comprehensive network of known protein interactions. For the Pathway Commons database the SIF interactions INTERACTS–WITH and STATE–CHANGE were taken into account (c.f. http://www.pathwaycommons.org/pc/sif-interaction-rules.do) and any self loops removed. For retrieval and merger of KEGG pathways we employed the R-package KEGGgraph [35].

In the resulting network graph (13,840 nodes with 397,454 edges) we have directed as well as undirected edges. For example, a directed edge *A*→*B* could indicate that protein *A* modifies protein *B* (e.g. via phosphorylation). An undirected edge *A*−*B* implies a not further specified type of direct interaction between *A* and *B*. Nodes in this network are identified via Entrez gene IDs. Genes in gene signatures can be thus be mapped to our protein interaction graph. Genes, which cannot be mapped, are not considered further.

We also investigated to expand this network further by putative transcription factor (TF)-target gene interactions, but our simulation study (see below) in this case clearly revealed an inferior performance compared to using a protein-protein interaction network only. This is probably due to the high number of false positives among inferred TF-target associations. Details can be found in the Supplement S1 to this paper (Figure S2).

**Algorithm 1** Pseudocode for a network based consensus signature.

ConsensusSignatures(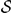,*G*,*k*)

Input:


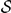
  =  set of gene signatures

*G*  =  partially directed graph (protein interaction network)

*k*  =  path distance cutoff (here 2)

consensus ← genes appearing in >50% of all signatures

candidates ← 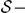
{consensus}

*D* ← shortest path distance matrix between candidates (ignoring edge directions)

*H* ← complete linkage clustering tree of candidates w.r.t. *D*

for *h* ←1…maximal pathway distance:

cut *H* at height *h* and obtain clusters


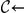
 clusters contaning genes from more than one signature

for each 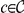:

consensus ← consensus 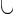 LCA(*c,k*) # note that the LCA can be empty

return consensus

### Network Based Consensus Signatures

#### Algorithm

Let 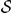 be a set of gene signatures and *G* be a partially directed graph (protein interaction network). A *k*-common ancestor (*k*-CA) of a set of nodes 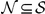 is defined as that set of nodes in *G*, from which all nodes in 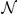 can be reached within path distance *k*. Each 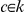-CA has thus a particular shortest path distance to each 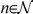, which is at most *k* (here *k* = 2). We define the LCA of 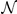 (abbreviated as 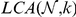) as a subset of the *k*-CA. More specifically, each 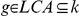-CA has to fulfill the additional criterion that the average shortest path distance to all nodes in 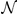 is minimal. Having this defined our algorithm works as described in pseudo code 1. The main idea is to successively look for groups of genes stemming from different signatures, which are close with respect to their shortest path distances in the network. For each such group we look for the LCA. Please note that the LCA does not need to be a non-empty set, because we have a defined path distance cutoff *k*. The likelihood of the LCA being empty obviously increases with higher spread of genes over the network.

Groups of genes are identified here by cutting a complete linkage clustering tree at a particular height. The choice of complete linkage clustering guarantees that we tend to get compact groups of approximately equal diameters (i.e. maximal shortest path distances between any pair of genes) [36]. This makes it more likely to find an LCA, which may show a certain regulatory influence on downstream genes.

The overall aim of our algorithm is to identify a consensus signature, which is enriched for genes being associated with the disease. Our hypothesis here is that LCAs together with genes appearing in the majority of gene signatures (“majority signature”) are good candidates for such a purpose.

The R-implementation of the proposed algorithm is part of the Supplements to this paper (code S1).

#### Performance Study

**Figure 7:**
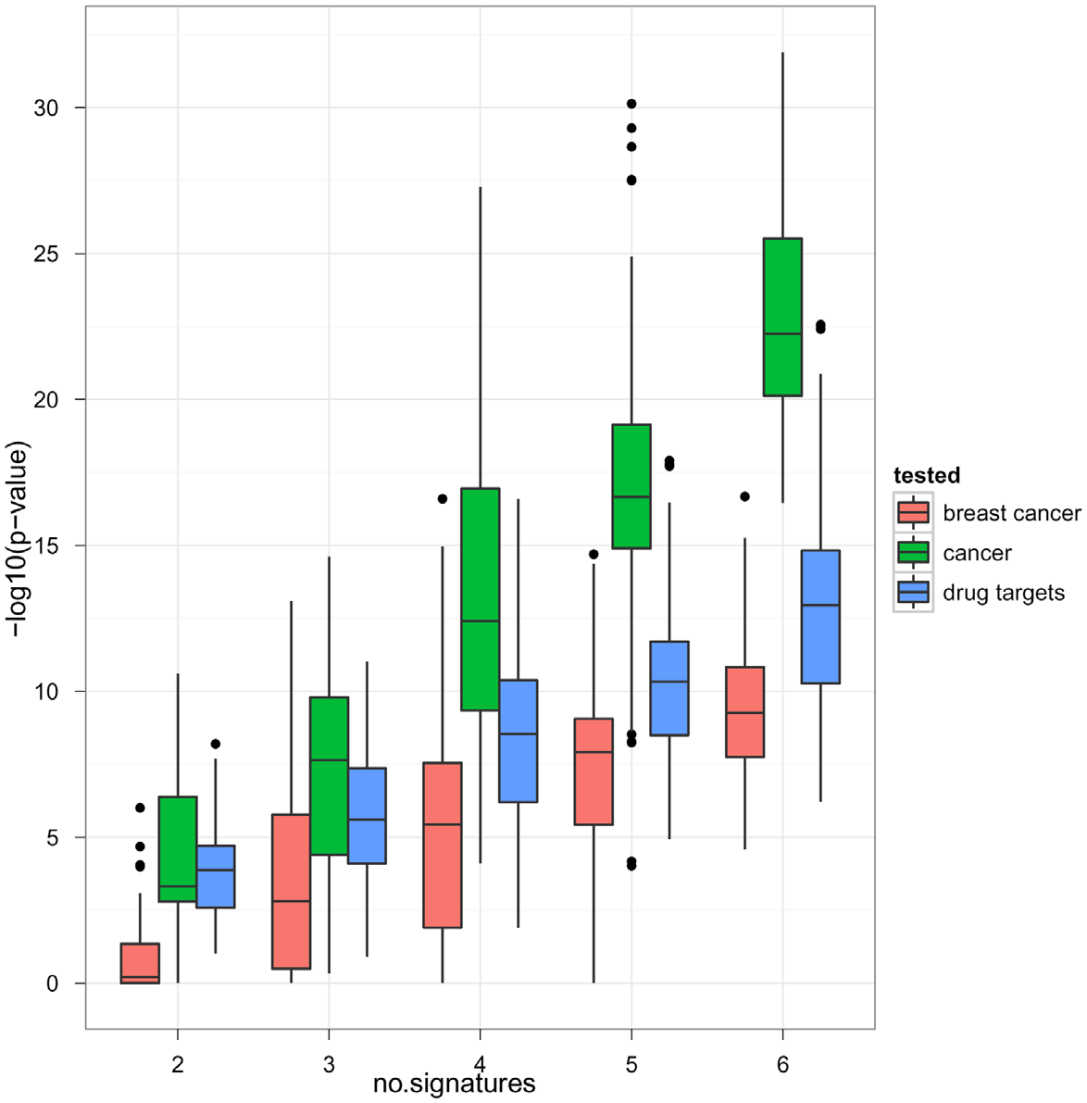
Enrichment of disease associated genes and drug targets in dependency on the number of gene signatures considered for a consensus.

We investigated the principal performance of our algorithm in terms of enrichment of disease associated genes and known drug targets in dependency on the number of gene signatures considered for the consensus. For this purpose from the six above described gene signatures we randomly picked 2,3,…,6 ones without replacement. For the picked gene signatures we then ran our algorithm and looked for significant enrichment of disease associated genes and drug targets. The procedure was repeated 100 times. This revealed a clear increase of significance in dependency on the number of gene signatures considered for a consensus (Figure 7), which implies that our algorithm picked up more and more disease relevant information. We would like to point out that not only the enrichment of cancer, but specifically also of breast cancer related genes increased with the number of used gene signatures. This means that not only the sensitivity, but also the specificity of the consensus signature was improved the more gene signatures were considered.

**Figure 8:**
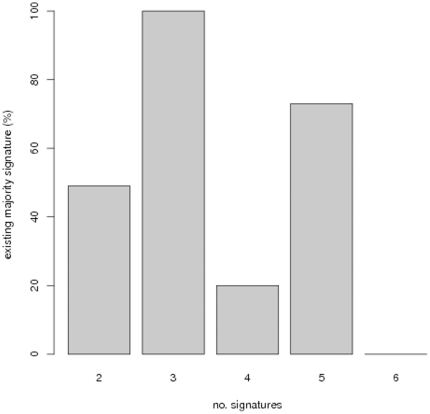
Fraction of times that a majority signature could be computed in dependency on the number of signatures.

**Figure 9:**
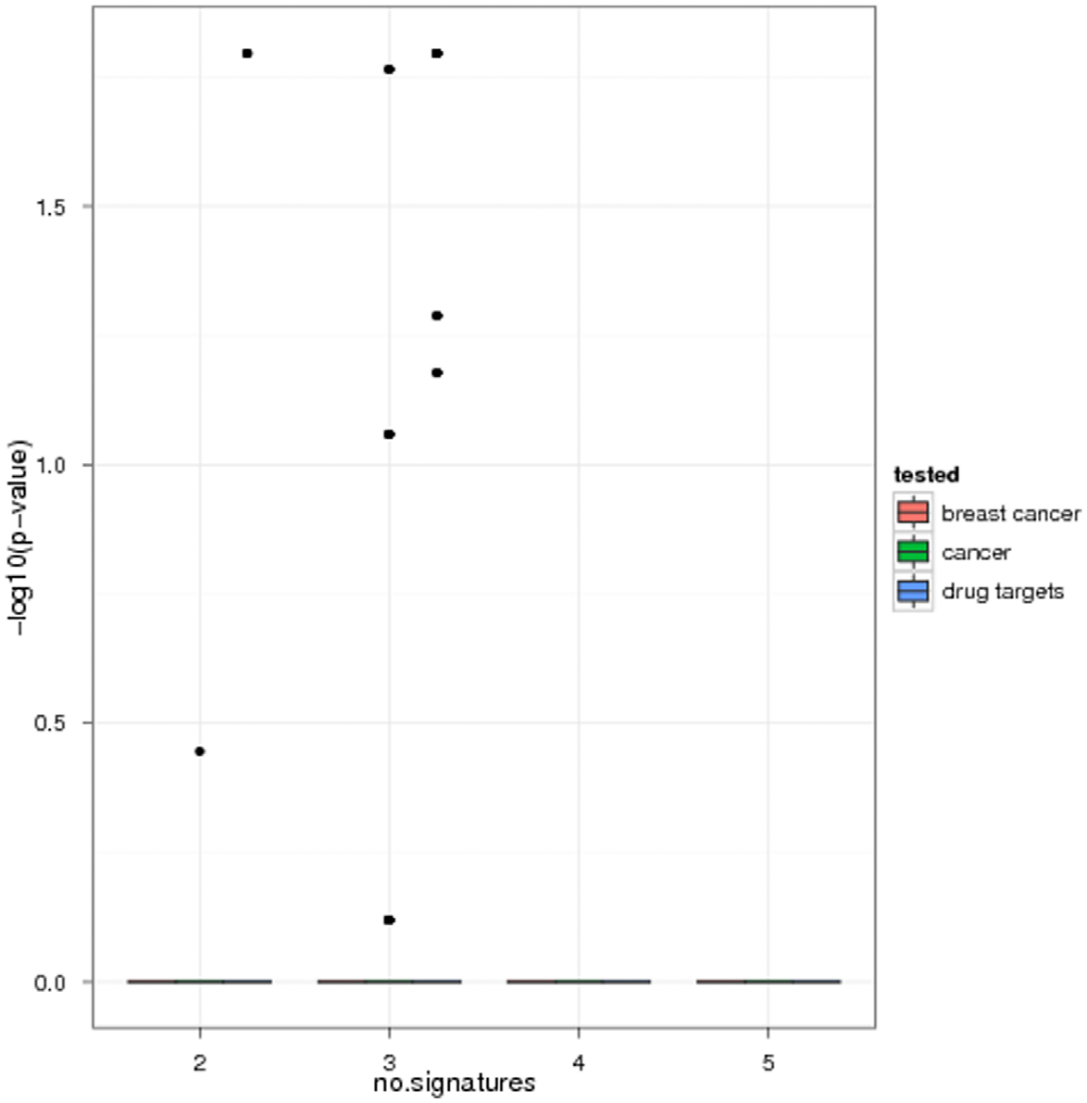
Majority signature: enrichment of disease associated genes and drug targets in dependency on the number of gene signatures. The enrichment was computed only, when the majority signature existed.

The existence of the majority signature, i.e. the signature containing only genes appearing in >50% of all compared signatures, varied greatly and with no clear trend depending on the number of compared signatures (Figure 8). Interestingly enough, in cases, where a majority signature could be established, it generally did not show any enrichment of disease associated genes and drug targets (Figure 9). This suggests that even in cases, where it is possible to compute a majority signature, our algorithm offers an additional benefit.

### Significance of Overlap

The significance of an overlap between a set of *n* signatures was determined via a random permutation test: We sampled 100,000 times signatures of the same size as the original ones from all unique Entrez gene IDs available on the HGU133A chip. Each time the size of the overlap was determined and in the end counted, how often the overlap of randomly sampled signatures exceeded the overlap of the original signatures. This yielded an empirical p-value, which was further corrected via the method by Phipson and Smyth [37].

## Supporting Information

Supplement S1 **Further details on simulation studies, retrieval of known drug targets construction of a TF-target network.**
(PDF)

Figure S1 **Probeset selection stability with random signatures of size n = 20 (left) and n = 50 (right).**
(TIFF)

Figure S2 **Enrichment of disease associated genes and drug targets in dependency on the number of gene signatures considered for a consensus:** Additional inclusion of TF-target gene associations.
(PDF)

Table S1 **Consensus signature.**
(XLS)

Table S2 **Results of FunDO analysis.**
(XLS)

Table S3 **Results of KEGG analysis.**
(XLS)

Table S4 **Information on known drug targets retrieved from MetaCore** **™.**
(XLS)

Table S5 **Predictive signature for the Ivshina et al. dataset.**
(XLS)

Table S6 **Predictive signature for the Mainz dataset.**
(XLS)

Table S7 **Predictive signature for the Pawitan et al. dataset.**
(XLS)

Table S8 **Results of FunDO analysis for Ivshina, Mainz and Pawitan datasets.**
(XLS)

Code S1 **R-implementation of pseudo-code.**
(R) Click here for additional data file.
