# Supplementary material for: Network Based Consensus Gene Signatures for Biomarker Discovery in Breast Cancer
Source: PLoS One. 2011 Oct 25;6(10):e25364. doi: 10.1371/journal.pone.0025364 (PMC3201953; doi:10.1371/journal.pone.0025364)
Supplement: Supplement S1 — Further details on simulation studies, retrieval of known drug targets construction of a TF-target network. (PDF) [file pone.0025364.s001.pdf]

Supplements: Network Based Consensus Gene Signatures for Biomarker Discovery in Breast Cancer

Holger Fröhlich

September 8, 2011

1 Simulation Study: Effect of Restricting the Set of Genes/Probesets on Selection Stability

We here exemplify the principal effect of a pre-selection of a set of genes/probesets on gene selection stability in a simulation study: Random signatures of n probesets ($n = 20, 50$) were either drawn from the set of all probesets on the HGU133A chip (whole array) or only sampled from an a-priori (arbitrarily) selected set of 100 probesets. This procedure was repeated 100 times. Figure 1 clearly shows in both cases a significant benefit by an a-priori restriction of probesets.

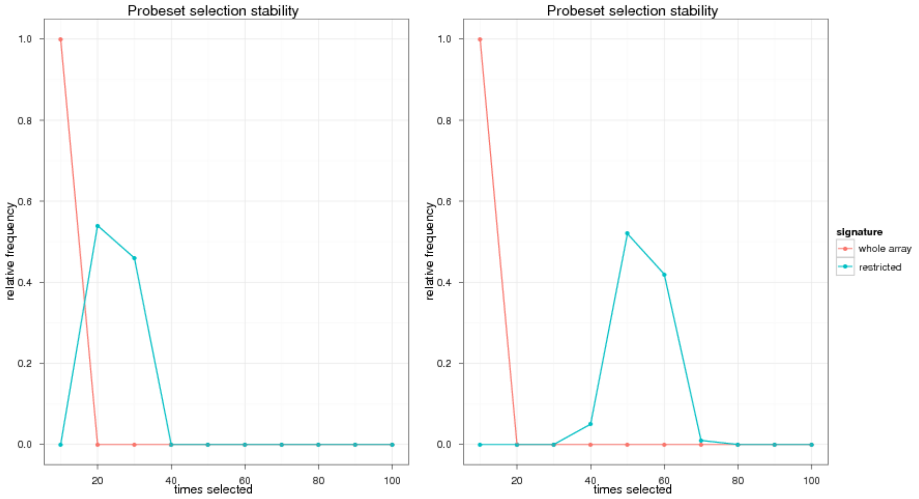

Figure 1: Probeset selection stability with random signatures of size $n = 20$ (**left**) and $n = 50$ (**right**).

Table 1: Enriched TF binding sites in each signature: #targets is reported as the number of unique Entrez gene IDs per individual TRANSFAC matrix.

Signature	Matrix	#targets
Li et al.	EN1, HOXA3	28
Bertucci et al.	-	-
Huang et al.	HEN1, OLF1, NRSF	142
van't Veer et al.	-	-
Sotiriou et al.	AP2alpha, E2F_03, E2F_02, NFY, SP1, SP1_Q6, ATF, STAT3, TAX/CREB	218
Wang et al.	FOXO4	74

2 Retrieval of Known Targets for Therapeutic Compounds

We looked for all proteins that are known targets of compounds (on the market, FDA approved or in clinical trials) against breast neoplasms or ductal breast carcinomas. The corresponding information was retrieved with the help of the software MetaCore^{TM}using the in-built search engine. The software MetaCore^{TM}provides access to a large, manually curated database of known protein & gene interactions as well as to known therapeutic compounds and their targets.

3 TF-Target Gene Network

3.1 Construction

For each of the six signatures considered here we looked for enriched transcription factor binding sites (TFBS) using the web interface of the software Pscan [Zambelli et al., 2009]. As source for position weight matrices we employed TRANSFAC here, and binding sites were searched in the range -1000bp till transcription start site. Only TFBS with a Bonferroni corrected p $<$ 5% were further taken into account. Those promoter gene sequences, in which Pscan reported an occurrence of a TFBS were considered as targets of the corresponding TF (see Table 1) and converted from REFSEQ into Entrez gene IDs. TRANSFAC matrix names were then mapped to Entrez gene identifiers in our protein-protein interaction network and a link from the corresponding TF to each of its putative target genes inserted. In cases, in which the mapping was not undoubtfully and uniquely possible, we decided to insert an extra node for each of these TRANSFAC matrices. This affected E2F, ATF, CREB and NFY. In consequence our combined PPI and TF-target gene network now had 13,918 nodes and 399,525 edges.

3.2 Simulation Results

The same kind of simulation as described in Section "Performance Study" in the main document was executed. In general the same principal behavior as with using protein-protein interactions only was observed. However, the performance on average was not as good (Figure 2), probably due to a high fraction of false positive TF-target interactions.

References

Federico Zambelli, Graziano Pesole, and Giulio Pavesi. Pscan: finding over-represented transcription factor binding site motifs in sequences from co-regulated or co-expressed genes. *Nucleic Acids Res*, 37(Web Server issue):W247–W252, Jul 2009. doi: 10.1093/nar/gkp464. URL http://dx.doi.org/10.1093/nar/gkp464.

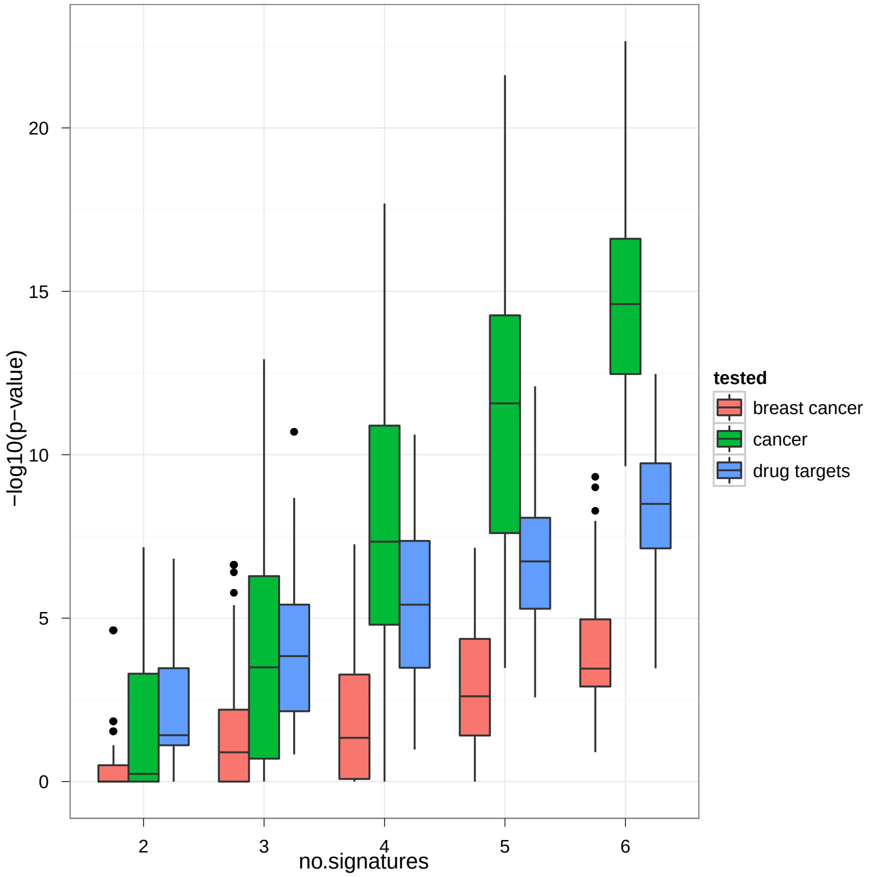

Figure 2: Enrichment of disease associated genes and drug targets in dependency on the number of gene signatures considered for a consensus: Additional inclusion of TF-target gene associations.
