# Supplementary figures and images for: Network Based Consensus Gene Signatures for Biomarker Discovery in Breast Cancer
Source: PLoS One. 2011 Oct 25;6(10):e25364. doi: 10.1371/journal.pone.0025364 (PMC3201953; doi:10.1371/journal.pone.0025364)

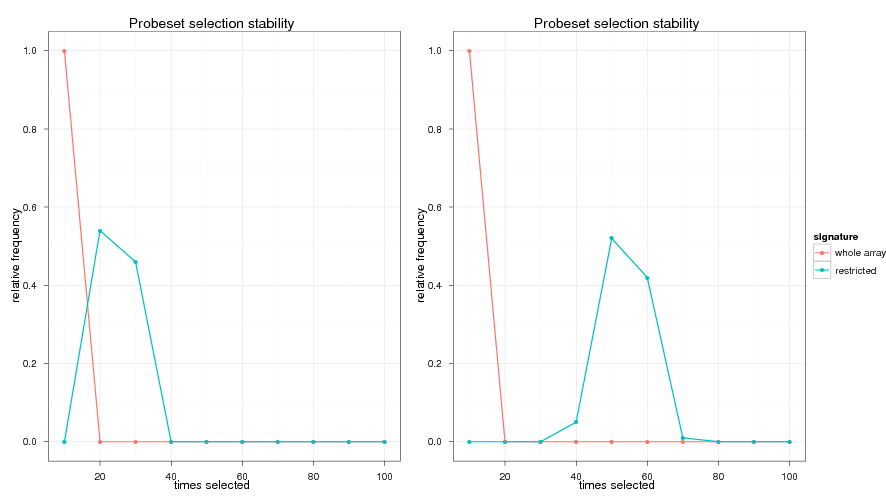

Supplement: Figure S1 — Probeset selection stability with random signatures of size n = 20 (left) and n = 50 (right). (TIFF) [file pone.0025364.s002.tif]

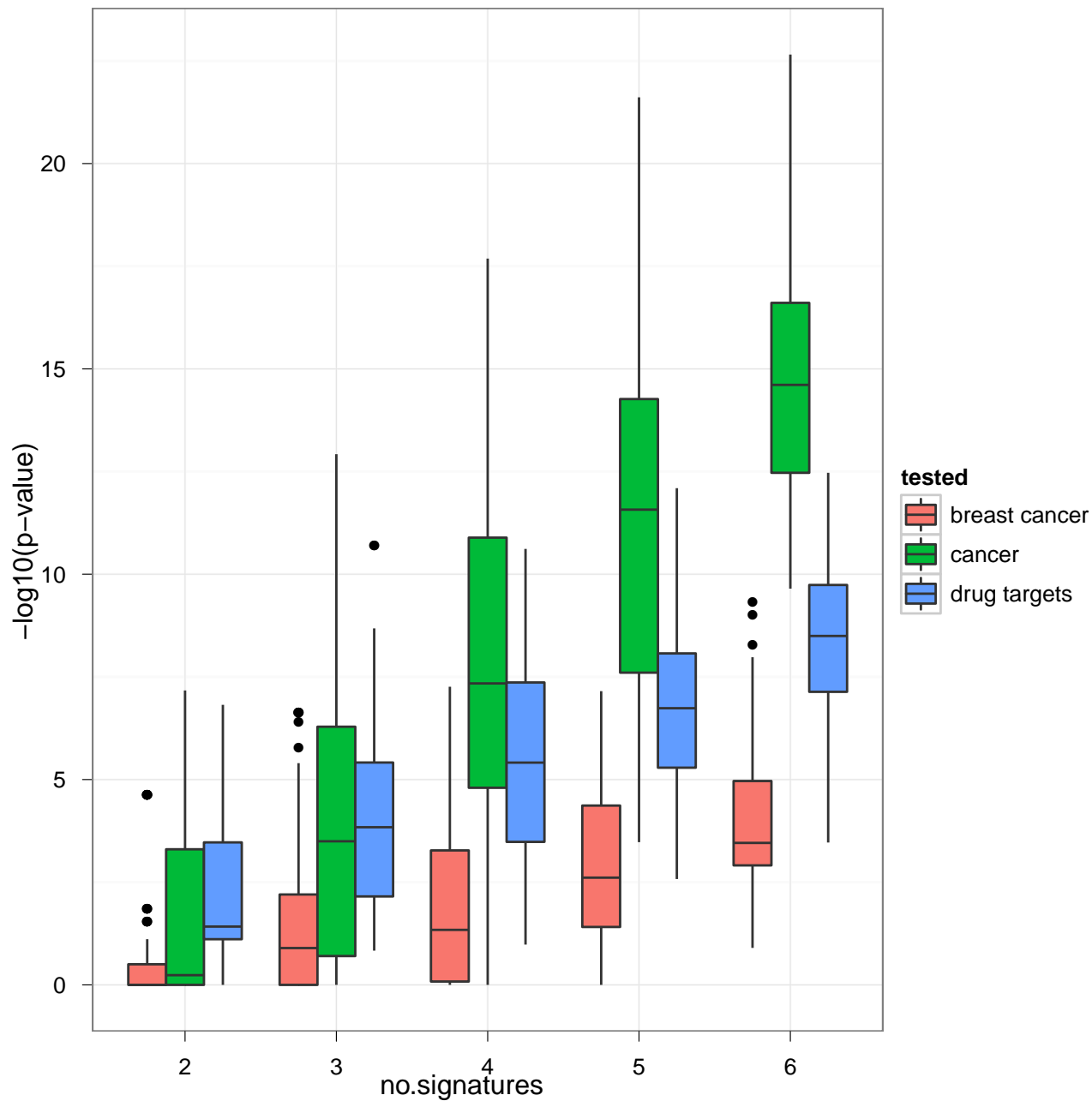

Supplement: Figure S2 — Enrichment of disease associated genes and drug targets in dependency on the number of gene signatures considered for a consensus: Additional inclusion of TF-target gene associations. (PDF) [file pone.0025364.s003.pdf]
